# Alone but flowing: The effects of autotelic personality and extraversion on solitary flow

**DOI:** 10.1111/jopy.12938

**Published:** 2024-05-10

**Authors:** Dwight C. K. Tse, Ayodele Joseph, Kate Sweeny

**Affiliations:** ^1^ Department of Psychological Sciences and Health University of Strathclyde Glasgow UK; ^2^ Department of Psychology University of California, Riverside Riverside California USA

**Keywords:** flow experience, flow state, interactive flow, solitude, solo flow

## Abstract

**Objective/Background:**

Flow, a psychological state of intense engagement in and enjoyment of an activity, can arise during both solitary and socially interactive experiences. In the literature, whereas people high in extraversion have difficulty achieving flow in solitude, those with an autotelic personality—a combination of traits that make people prone to flow—readily experience flow in both solitary and interactive conditions. In this pre‐registered experiment, we investigated whether autotelic personality mitigates the negative association between solitary flow and extraversion.

**Method:**

Participants and their romantic partners (final *N* = 368) played the game Perfection™ in three conditions (order was counterbalanced): alone (*solitary condition*), in the presence of their partner without interaction (*mere‐presence condition*), and collaboratively (*interactive condition*).

**Results:**

There were independent, positive main effects of extraversion and autotelic personality on flow experience in mere‐presence and interactive conditions. However, the positive effect of extraversion on solitary flow was only significant among participants with high (vs. low) autotelic personality. In all conditions, flow experience was associated with greater low‐arousal positive affect and lesser high‐arousal negative affect.

**Conclusions:**

The findings shed light on the role of personality in promoting solitary flow experiences, and particularly how traits might interact to determine optimal and non‐optimal conditions for achieving flow.

## 1 INTRODUCTION

One path to a flourishing and thriving life is staying engaged in many activities. Flow experience is a psychological state of engagement characterized by intense concentration and enjoyment (Csikszentmihalyi, 1990). In the flow state, people experience a heightened sense of control, complete concentration, merging of action and awareness, loss of self‐consciousness, distorted time perception, and autotelic experience (an experience that is worth pursuing on its own), which is usually facilitated by conditions including a balance between challenges and skills, proximal goals, and unambiguous feedback (Nakamura et al., 2019). Studies have demonstrated that prolonged engagement in flow‐conducive activities is associated with better performance, greater motivation, and better well‐being outcomes (see Landhäußer & Keller, 2012 for a review).

Research interest in solitary flow experience (i.e., in the absence of social interaction; Larson, 1990) has been surprisingly scarce compared to that in social contexts, as depicted by the plethora of research on team flow, group flow, shared flow, interactive flow, and flow contagion in the literature (e.g., Bakker, 2005; Sawyer, 2014; van den Hout et al., 2018). Investigating flow experience in solitude may aid our understanding of the benefits of being alone. Theories and empirical studies have differentiated positive (or benign) solitary experiences from negative ones, with key factors in beneficial experiences including a sense of control, freedom from distractions, and engagement in creative and/or self‐transcendent (e.g., spirituality‐related) activities (Long & Averill, 2003). These experiences appear to be closely related to the flow state, suggesting that at least some moments when people enjoy solitude are also the time when they experience flow in their solo activities.

In this Stage 2 Report, we presented the findings from a pre‐registered experiment examining flow experiences in solitary and interactive settings. Whereas there has been accumulating evidence of some personality traits that are conducive to flow experience, it is unclear whether such effects are sensitive to social contexts. As such, we specifically investigated relationships among autotelic personality (a constellation of dispositions that facilitate flow experience across domains; Baumann, 2021), extraversion, and flow experiences in the same activity in solitude or with social interaction. The findings would help differentiate personality traits that have context‐specific and context‐general effects on flow experience and test whether these dispositions may interact in different social contexts.

### 1.1 Solitary and interactive flow

Since the conceptualization of flow experience in 1975, theorists have been debating the differences between the flow state experienced alone versus when engaging in interactive activities (e.g., Magyaródi & Oláh, 2015; Sawyer, 2014). Despite great interest in understanding the flow state when people engage in activities involving others, solitary or solo flow remains a subsidiary part of the “typical flow experience” with little interest in its unique predictors and outcomes. It is questionable the extent to which the research findings on typical flow experience can be assumed to be applicable to flow experience in solitude, given that many flow‐conducive activities are interactive in nature (e.g., team sports and band music; Bakker et al., 2011; Sawyer, 2014). Conflating solitary and interactive flow leads to imprecise estimates of the directions and magnitudes of the factors predicting each kind of flow experience.

Walker (2010, see also Walker, 2021) conducted one of the first few studies that experimentally compared solitary and interactive (social) flow, in which participants engaged in experimental tasks such as paddleball and pickleball games. Depending on the operationalizations of flow experience, the findings revealed no consistent differences in flow between the dyadic (volleying a ball between partners) and the solitary conditions (bouncing a ball off a wall), or between a highly interdependent task and a less interdependent one. Nevertheless, combined with the survey findings on solitary and interactive flow in everyday life, Walker highlighted the uniqueness of solitary flow experiences concerning conditions and indicators. For example, task feedback in solitary flow is primarily cognitive, whereas the social feedback in interactive flow is primarily affective.

However, Walker's (2010) experiments did not consider dispositional factors that facilitate solitary and interactive flow experiences. Although experimental findings suggest that certain dispositions, such as internal locus of control and high action‐orientation (Keller & Bless, 2008; Keller & Blomann, 2008), facilitate flow experiences even when the situation is not flow‐conducive, these experimental studies employing the single‐player Tetris™ paradigm did not include an interactive task for comparison. Whether these dispositional effects were specific to solo tasks remains an open question. In another experiment that examined flow experiences in squared puzzle tasks played solo or collaboratively (Tse et al., 2018), the effect of flow proneness, or the tendency to experience flow in daily life, on flow experience did not interact with the nature of the task (solo or collaborative). Taken together, there is a paucity of experimental studies directly comparing flow experiences in solitude or interaction, making it challenging to conclude which personality traits facilitate flow experiences in solitude per se.

### 1.2 Personality and solitary flow

Personality research on flow experience investigates how individual differences in dispositions can reliably predict differences in flow experiences (Baumann, 2021). Csikszentmihalyi et al. (1997) coined the term *autotelic personality* referring to dispositions that generally facilitate flow experiences across life domains and activities. Recently, researchers developed a self‐administered questionnaire to measure seven core autotelic dispositions, namely curiosity, persistence, low self‐centeredness, intrinsic motivation, enjoyment of challenges, transformation of boredom, and attentional control (Tse, Lau, et al., 2021). As predicted, autotelic personality is associated positively with flow proneness across life domains and has a positive effect on everyday flow experience in diary and experience sampling studies (Asakawa, 2010; Tse, Nakamura, et al., 2021; see also Baumann & Scheffer, 2010 for the relationship between flow experiences and autotelic personality operationalized as achievement flow motive). Although studies of autotelic personality have not distinguished its predictive power on the flow state in solitary versus interactive activities, based on its theoretical definition (i.e., predicting flow across contexts), we hypothesized that autotelic personality has a facilitating effect on both solitary and interactive flow experiences (*Hypothesis 1*).

As a dispositional construct that is closely tied with flow experience, autotelic personality has received relatively little attention in the broader personality literature. However, autotelic personality has important implications for life engagement and well‐being. Asakawa (2004) found that autotelic college students were more likely to place themselves in slightly challenging situations in comparison with their skill levels, which arguably provided them with more opportunities for action and led to personal growth and mastery (Massimini & Delle Fave, 2000). In another study, Japanese college students with high autotelic personality showed greater commitments to college life and more active search for future careers than their counterparts (Asakawa, 2010). They also reported higher self‐esteem, lower anxiety, and a greater sense of *Jujitsu‐kan—*a sense of life fulfillment unique in Japanese culture.

Besides Japanese college students, the impact of autotelic personality predicting long‐term academic success was also evident across various cultures (Busch et al., 2013), perhaps due to the tendency of those with autotelic personalities to seek and master challenges (Baumann, 2021). Beyond success in specific domains, people with high (vs. low) autotelic personality are indeed more likely to enter the flow state in everyday activities (Johnson et al., 2014) and, in turn, enjoy a flourishing life with more positive affect and higher satisfaction with life (Tse, Nakamura, et al., 2021). Given that many flow‐conducive activities can take place in solitary contexts (e.g., playing sports and music alone), examining autotelic personality may help unveil dispositions that are conducive to positive solitude, above and beyond traits that are typically related to social situation preferences such as extraversion.

Personality research that directly compares flow experiences in solitary and interactive activities is scarce. While many studies have revealed an overall positive relationship between extraversion and general flow experiences (irrespective of social contexts; see Peifer et al., 2022 for a review; cf. Johnson et al., 2014; Ullén et al., 2012), Liu and Csikszentmihalyi (2021) conducted a survey study using a recall approach to investigate differences in flow experiences between solitary and interactive activities. Their findings revealed that extraverts enjoyed more intense and frequent flow experiences in social activities, whereas introverts perceived solitary activities to be more flow‐conducive than interactive activities, suggesting that the extraversion–flow relationship may differ across social contexts.

Because high levels of extraversion denote sociability and assertiveness, highly extraverted individuals are more likely to dominate the kind of activities they engage in with others, leading to their greater tendency to experience flow in interactive contexts than those low in extraversion. In contrast, extraverts (vs. introverts) may find that solitary activities lack sufficient stimulation from the social environment, leading to the experience of boredom that is antagonistic to the flow state. In the solitude literature, extraversion is negatively associated with the preference and capacity for solitude (e.g., Burger, 1995; Hills & Argyle, 2001; Lin et al., 2020; cf. Nguyen et al., 2022, Zelenski et al., 2013), which may lead to a perception of the solitary context as suboptimal for them to fully engage in activities. Taken together, we hypothesized that extraversion has an inhibitory effect on solitary flow and a facilitating effect on interactive flow (*Hypothesis 2*).

One underexplored area is how the interaction between personality traits may contribute to differential flow experiences in solitary and interactive activities. Extrapolating from person–environment fit theory (e.g., Caplan & van Harrison, 1993; Edwards et al., 1998), we posit a misfit between people high in extraversion and a solitary context, creating a suboptimal experience and difficulty in achieving the flow state in solitude (see also Moneta, 2012). Although the mechanism of how people high in autotelic personality achieve frequent flow experiences remains unclear to date, studies have found that flow proneness can mitigate the impact of a suboptimal environment on flow experiences (e.g., when the challenge level is too high; Tse et al., 2018). This finding suggests that autotelic personality may be accompanied by the ability to transform boring (under‐stimulating) or highly anxious (over‐stimulating) situations into flow‐conducive ones. As such, although extraverts may not find the solitary context an ideal environment in which to achieve the flow state, we hypothesized those who are also high in autotelic personality are less affected by the person–environment misfit and maintain high levels of flow experiences in solitude (*Hypothesis 3*).

### 1.3 The current study

This study examined the relationship between personality factors and flow experience across social contexts. We conducted an experiment that controlled for potential confounding factors. First, we employed a within‐person design in which participants worked on the same experimental task alone and with a close other. In Liu and Csikszentmihalyi's (2021) recall prompt design, there was no restriction concerning the type of solitary or interactive activities people recalled, and the flow scales were tailored such that they asked about the *general* intensity and frequency of flow experiences in each social context. Walker (2010) adapted the experimental tasks differently between solitary (bouncing a ball to the wall) and interactive conditions (bouncing a ball between partners), confounding the comparisons between conditions as the tasks draw on different skills (e.g., bouncing a ball between partners required observations and anticipations of another player's actions). Using the same experimental task provided a common ground for fair comparisons, such that the only difference between solitary and interactive conditions would be the presence/absence of social interaction.

Another factor to control was the interactive partner. In this study, we recruited romantic partners in a stable relationship. This approach was similar to Graham's (2008) experience sampling study on couples' momentary flow experiences, although Graham did not compare solitary experiences directly against interactive experiences with a romantic partner. Compared to friends, acquaintances, or even strangers (Tse et al., 2018), romantic partners are more prone to having high‐quality social exchanges. Studies have also revealed that they are one of the social targets with whom adults tend to spend the most time interacting in everyday life (Ortiz‐Ospina et al., 2020). As an active control, we included a condition in which participants were instructed not to communicate with each other while they work individually on the same task in the same room (i.e., a mere‐presence condition). Romantic partners, especially those who are cohabiting, are also more likely to be involved in this type of presence‐without‐interaction environment. We anticipated that the recruitment of romantic partners would facilitate a high volume of exchange in the interactive condition and contribute to experimental realism, as opposed to experimentally imposing communications between two unrelated strangers.

Finally, although our study focused on personality predictors of solo flow, another area to explore was the downstream implications of flow experiences. Specifically, we anticipated that across conditions, greater flow experience would be associated with more intense high‐ and low‐arousal positive affect and greater motivation to reengage in the task (*Hypothesis 4*). In the solitude literature, solitude was considered a self‐regulatory mechanism for downregulating high‐arousal emotions (e.g., Nguyen et al., 2018), and such a downregulation process appears to be more beneficial for specific groups, such as older adults (Pauly et al., 2017). Nevertheless, it is noteworthy that these findings are based on experimental conditions in which participants were instructed not to engage in any tasks, read leisurely, or think about something in solitude; or diary or experience sampling designs in which participants engaged in different activities across solitary and social situations. That approach differed from our proposed study, in which we asked participants to engage in an absorbing, flow‐conducive activity. In the flow literature, engagement in flow‐conducive activities is associated with heightened high‐arousal (e.g., excitement) and low‐arousal (e.g., serene) positive affect, although previous studies did not differentiate solo vs. interactive flow (e.g., Jiang et al., 2024; Tse, Nakamura, et al., 2021). We posit that in contrast to socially oriented positive affect such as feeling respected or connected with others, general positive affect (as well as general motivation to reengage) should have a similar association with flow state during the activity, regardless of the social (or asocial) contexts. Together with the previous discussion on the personality predictors of solo flow, such an investigation may contribute to a better understanding of how people with various dispositions enjoy and sustain engagement in flow‐conducive activities when alone (Hypothesis 4). It would also contribute to the solitude literature by revisiting the downregulation hypothesis, with the solo activity being experimentally controlled as absorbing and intrinsically enjoyable.

Taken together, we tested the following hypotheses in the experiment.

Autotelic personality is associated positively with flow experience in solitary, mere‐presence, and interactive conditions.

Extraversion is associated with flow experience negatively in the solitary condition but positively in the mere‐presence and interactive conditions.

Autotelic personality interacts with extraversion, such that the negative effect of extraversion on solitary flow (but not flow experience in mere‐presence or interactive conditions) is weaker among people with high (vs. low) autotelic personality.

Across conditions, flow experience is associated with greater high‐ and low‐arousal positive affect and greater motivation to reengage in the task.

## 2 METHOD

All study data and data analysis codes are available at https://osf.io/pqx3z/?view_only=767a9d5bcc0d4bf09b4dab6c70d2a135 (also including the Stage 1 Registered Report) and in the special issue's Open Science Framework (OSF) repository.

### 2.1 Participants

We recruited 396 (see Table 1 for demographic information) undergraduate participants from the highly diverse Psychology Participant Pool at the University of California, Riverside, who had been in a romantic relationship for at least 6 months.^1^ Based on Liu and Csikszentmihalyi's (2021) findings on extraversion and solitary and interactive flow, we determined our smallest effect size of interest to be *r* = 0.18, and the corresponding target *N* = 190 (95 couples), with power = 0.80 and alpha = 0.05 calculated by G*Power (Faul et al., 2009). We planned to stop data collection when the sample size was at least 10% beyond the target for data quality assurance, but we ended up having more participants signed up for this study. The final sample size (*N* = 368, see below for exclusion criteria) is adequate to achieve power = 0.99, 0.97, and 0.92, for Δ*R* ^2^ = 0.05, 0.04, and 0.03, respectively, with alpha = 0.05 in a path analysis model. It is also adequate to detect the difference between a poorly fit model (root mean squared error of approximation [RMSEA] = 0.08) and a model with good fit (RMSEA = 0.05) with power = 0.91 and alpha = 0.05 (Preacher & Coffman, 2006).

**TABLE 1:** Descriptive statistics of final and excluded samples.

Variables	Final sample (*n* = 368)		Excluded sample (*n* = 28)		Cohen's *d* [95% CI]/*χ* ^2^(*df*)
	*M*/*n*	*SD*/%	*M*/*n*	*SD*/%	
Age	19.87	2.94	19.14	1.18	−0.33* [−0.53, −0.07]
Gender					1.87 (2)
Male	146	39.78	14	50.00	
Female	208	56.68	14	50.00	
Non‐binary	13	3.54	0	0.00	
Ethnicity					7.48* (2)
Asian	127	34.61	12	42.86	
Latinx	157	42.78	5	17.86	
Other ethnicities	83	22.62	11	39.29	
Game experience (1–4)	1.27	0.59	1.21	0.50	−0.11 [−0.44, 0.33]
Relationship satisfaction (1–7)	6.38	0.62	6.21	0.69	−0.26 [−0.67, 0.12]
Relationship length (months)	19.39	21.75	17.52	9.82	−0.11 [−0.36, 0.18]
Autotelic personality (1–7)	4.59	0.63	4.67	0.57	0.14 [−0.21, 0.52]
Extraversion (1–5)	3.23	0.78	3.25	0.70	0.04 [−0.35, 0.41]
Number of “wins” (solitary; 0–3)	1.45	1.16	1.96	0.88	0.50** [0.14, 0.85]
Number of “wins” (mere‐presence; 0–3)	1.50	1.17	1.71	1.18	0.18 [−0.21, 0.57]
Number of “wins” (interactive; 0–3)	2.11	0.93	2.36	0.73	0.30 [−0.08, 0.64]
Flow experience (solitary; 1–5)	3.95	0.65	4.15	0.52	0.33 [−0.04, 0.70]
Flow experience (mere‐presence; 1–5)	3.88	0.68	3.94	0.60	0.09 [−0.30, 0.44]
Flow experience (interactive; 1–5)	3.97	0.63	4.10	0.56	0.21 [−0.18, 0.58]
LAP affect (solitary; 1–5)	3.16	1.03	3.10	0.98	−0.05 [−0.44, 0.33]
LAP affect (mere‐presence; 1–5)	3.10	1.04	2.93	0.93	−0.18 [−0.57, 0.22]
LAP affect (interactive; 1–5)	3.40	0.97	3.48	0.99	0.08 [−0.32, 0.49]
HAN affect (solitary; 1–5)	1.68	0.63	1.61	0.61	−0.11 [−0.47, 0.35]
HAN affect (mere‐presence; 1–5)	1.72	0.66	1.82	0.63	0.16 [−0.20, 0.55]
HAN affect (interactive; 1–5)	1.52	0.54	1.58	0.76	0.09 [−0.35, 0.51]

*Note*: Confidence intervals are bootstrapped at 95% level.

Abbreviations: HAN, high‐arousal negative; LAP, low‐arousal positive.

*p < 0.05; ** *p* < 0.01.

### 2.2 Procedure

We conducted an experiment with three within‐person conditions (context: solo, interactive, mere‐presence) and two between‐person predictors (autotelic personality and extraversion). The study took place in a psychology lab at the University of California, Riverside in two separate rooms. Participants first completed a pre‐task survey on their personality and demographic information (all surveys were administered electronically). Then, participants played a board game called Perfection™ (Milton Bradley Company, 1989) in each of three conditions. The game required participants to place pieces of different shapes into matching holes on a play board before the time ran out. When the time limit was up, the play board sprung up, and the pieces flew out of the holes. In the *solo condition*, members of the couple worked on the task alone in separate rooms. The *mere‐presence condition* was an active control, in which participating couples worked on the tasks individually in the same room without any interaction. In the *interactive condition*, couples worked on the same task together, with instructions explicitly encouraging their communication during the task. To better balance the challenge level in the interactive condition given that two players worked on the same board, we instructed participants to put yellow pieces only in odd number rows and red pieces only in even number rows. To account for practice effects and boredom due to repetitions, the condition orders were counterbalanced using the Latin square design. Each dyad completed all conditions in one session.

In each condition, participants played the game three times; the pieces were reshuffled between each round. The time limit for each round was 60 seconds, such that typical players would be unable to complete the game to avoid the conflation of a sense of achievement and the flow state induced from the task (Bakker et al., 2011). In cases in which participants had completed the game before the time limit, we originally planned to run sensitivity analyses with and without these conditions. However, we discovered that there were more cases that had completed (“won”) the Perfection task before the time limit. For example, only 24 participants were unable to complete any of the three trials in the interactive conditions. Therefore, we retained all cases for analyses and included the number of completion (“wins”) in subsequent analyses as a covariate. We pilot‐tested the task to ensure that it would be flow‐conducive to most players.

We originally planned to video‐record the experimental sessions for data quality assurance. Nevertheless, due to unforeseen technical difficulties that we were unable to solve before data collection, we reverted to having experimenters blinded to the hypotheses making notes of any deviations from instruction, such as uninstructed communications between participants in mere‐presence conditions (operationalized as more than three verbal exchanges between participants), or the lack thereof in interactive conditions (i.e., fewer than three exchanges). Then, two authors (DT and AJ) who did not collect data independently determined the records to be included or excluded based on the experimenter notes. The pair achieved excellent inter‐rater agreement in their first attempt (Cohen's kappa = 0.884, *p* < 0.001) and resolved the discrepancies by discussion. Out of 396 participants, 28 were excluded from further analyses, leaving the final *N* = 368. Compared to the final samples, excluded participants were significantly younger, more likely to self‐identify as Asians, and more likely to complete (“win”) the game in the solitary condition. Table 1 shows the descriptive statistics of both excluded and final samples.

### 2.3 Measures

#### 2.3.1 Flow experience

After each condition, participants completed the 9‐item Short Flow State Scale (SFSS; Jackson et al., 2008) to indicate the extent to which they have experienced the nine dimensions of flow experiences (e.g., “The experience is extremely rewarding,” 1 = *strong disagree*, 5 = *strongly agree*). The SFSS has been validated and used extensively to capture the intensity of flow experience across activities, including experimental tasks (e.g., Harmat et al., 2015; Tse et al., 2018). The Cronbach's alphas were 0.77, 0.79, and 0.76 in solitary, mere‐presence, and interactive conditions, respectively.

#### 2.3.2 Autotelic personality

In the pre‐survey, we administered the 26‐item Autotelic Personality Questionnaire (APQ; Tse, Lau, et al., 2021) to measure the seven core facets of autotelic personality (e.g., “I enjoy playing difficult games,” 1 = *strongly disagree*, 7 = *strongly agree*). Higher average scores indicated greater autotelic personality. The Cronbach's alpha was 0.83.

There are concerns about the conceptual overlap between autotelic personality and flow experience, given the definition of the former construct (i.e., “dispositions that facilitate flow experiences across domains”). To address this, we compared the fit of two latent factor models with confirmatory factor analyses. Specifically, one model included autotelic personality and flow experience as separate factors. Another model had one overarching factor with autotelic personality dispositions and flow experiences across conditions loading on it. If autotelic personality is conceptually distinct from flow experience, the first model should show a better fit than another (see below for the model comparison information). Indeed, the overarching factor model demonstrated significantly worse fit (Δ *χ* ^2^[2] = 239.03, *p* < 0.001, ΔCFI = 0.304, ΔRMSEA = 0.075, ΔSRMR = 0.051), supporting the conceptual distinctiveness between autotelic personality and flow experience.

#### 2.3.3 Extraversion

We measured extraversion with the 8‐item subscale of the Big Five Inventory (John & Srivastava, 1999). Participants indicated the extent to which each statement, such as being “outgoing and sociable,” applied to them (1 = *disagree strongly*, 5 = *agree strongly*). Higher average scores reflected greater extraversion. The Cronbach's alpha was 0.85.

#### 2.3.4 Immediate affect

We originally planned to capture (a) high‐arousal positive affect and (b) low‐arousal positive affect with Tsai et al.’ (2006) scale, and (c) task motivation by Keller et al. (2011) “free choice” procedure. However, due to the research team's oversight, the final data collection materials did not include these measures. Instead, we only administered a 24‐item affective scale (Sweeny, 2023) measuring participants' high‐arousal negative (HAN) affect (e.g., “irritated,” “frustrated”) and low‐arousal positive (LAP) affect (e.g., “peaceful,” “calm”) immediately after the game using a 7‐point scale (1 = *not at all*, 7 = *extremely*). The scale did not enable us to test Hypothesis 4, but at the very least, it captured participants' affective states that could inform other exploratory analyses (see below). The Cronbach's alphas for HAN affect were 0.89 (solitary), 0.89 (mere‐presence), and 0.85 (interactive), and those for LAP affect were 0.96 (solitary), 0.96 (mere‐presence), and 0.95 (interactive).

#### 2.3.5 Demographic and relationship variables

We collected demographic and relationship information including participants' age, gender, experiences with the game Perfection™ (1 = *never played it before*, 4 = *played it very often recently*), relationship satisfaction (12‐item Perceived Relationship Quality Component Scale; Fletcher et al., 2000; 1 = *very dissatisfied*, 7 = *very satisfied*; Cronbach's alpha = 0.91), and their relationship length (in months). Because participants completed all conditions, we did not anticipate these variables to influence the within‐person comparisons. However, in case any of these variables showed a statistically significant relationship with flow experience in any conditions, we would include the variable(s) in our analyses as covariates to control for its impact on our findings.

### 2.4 Analysis plan

We first removed cases (*n* = 28; see Procedure) that deviated from the instructions listwise from the dataset. Before computing the composite scores for each scale, we examined their corresponding Cronbach's alphas. For alphas <0.70, we explored remedies such as the deletion of poorly performing items to enhance internal consistency. No Cronbach's alpha fell below 0.70. We inspected descriptive statistics and bivariate correlations among variables (see Table S1). Based on the correlation findings, we included age, Perfection™ game experience, relationship satisfaction, and number of “wins” as covariates in subsequent analyses. We did not eliminate univariate or multivariate outliers as they could contain potentially meaningful patterns (see below for the bootstrapping procedure).

We tested the hypotheses by estimating a path model with the covariates (see above), autotelic personality (H1), extraversion (H2), and their interaction term (H3) as predictors. We included flow experiences in solo, mere‐presence, and interactive conditions as parallel endogenous variables in the model. We also intended to examine the downstream relationships among flow experiences, HAP and LAP affect, and task motivation (H4). We were unable to test this hypothesis fully due to some scales being mistakenly excluded from the data collection materials. Instead, we were able to investigate the downstream relationships among flow experiences and HAN and LAP affect. Such investigation still contributes to the literature and provides insights about the downregulation effect of solitude (see Discussion).

For all analyses, we mean‐centered the predictors and created the interaction term by multiplying the centered variables. The mean‐centered interaction term can address the multi‐collinearity issue (James et al., 2017). We determined an adequate model fit with comparative fit index (CFI) >0.90, standardized root mean square residual (SRMR) <0.08, and RMSEA <0.08 (Hu & Bentler, 1999). We evaluated effect sizes based on *R* ^2^ and standardized regression coefficients of corresponding predictors. We compared the model fit between a model that constrained the effect of personality on flow experience to be equal across conditions and an unconstrained counterpart. We also conducted model comparisons to investigate whether the effect of flow experience on each outcome variable would be significantly different across conditions. Using the chi‐squared difference test, if the unconstrained model demonstrates a better fit with the data than the constrained model, we considered the regression coefficients to be significantly different across conditions. We estimated the indirect effects of autotelic personality and extraversion on HAN and LAP affect through flow experience by multiplying the effect of personality on flow experience (path a) and that of flow experience on affect and motivation (path b). Additionally, if the interaction between autotelic personality and extraversion was significant (H3), we followed up by conducting simple slope analyses and estimated the conditional indirect effects.

Although we did not anticipate serious missing data issues because all data were collected on‐site, we handled missing data using the full‐information maximum likelihood estimation. To account for the potential deviation from the parametric assumptions, we conducted all hypothesis testing with a bootstrapping procedure with at least 5000 resamples. For each data deletion step and any unplanned statistical decisions, we conducted sensitivity analyses to evaluate the robustness of the findings by comparing them with or without the deleted data and/or the alternative statistical options. We conducted all statistical procedures in R, using packages such as lavaan and semTools. Without a meta‐analysis on the relationship between personality and flow experience in the literature, we interpreted the effect size based on the conventional cutoffs with *R* ^2^ = 0.02, 0.13, and 0.26 as indications of a small, medium, and large effect, respectively (Cohen, 1988). We determined *p* < 0.05 as an indication of statistical significance.

## 3 RESULTS

### 3.1 Planned analyses

The path analysis model demonstrated good model fit, CFI = 0.974, RMSEA = 0.061, 90% CI [0.045, 0.078], and SRMR = 0.046. Table 2 shows the standardized estimates and their 95% CIs. First, consistent with H1, autotelic personality was positively associated with flow in solitary, mere‐presence, and interactive conditions. Constraining the paths across conditions to be equal did not significantly worsen the model fit (Δ*χ* ^2^[2] = 0.34, *p* = 0.842, ΔCFI = 0.001, ΔRMSEA = 0.003, ΔSRMR = 0.000), suggesting that the main effects of autotelic personality on flow experience were highly similar across conditions.

**TABLE 2:** Standardized estimates [95% confidence intervals] of the path analysis model.

Predictor	Alone			Mere presence			Interactive		
	Flow	LAP	HAN	Flow	LAP	HAN	Flow	LAP	HAN
Autotelic personality (AP)	0.12* [0.02, 0.23]	0.11* [0.01, 0.21]	−0.02 [−0.12, 0.07]	0.14** [0.04, 0.24]	0.17** [0.07, 0.27]	−0.09 [−0.20, 0.02]	0.16 ** [0.05, 0.26]	0.14* [0.03, 0.25]	−0.01 [−0.14, 0.13]
Extraversion (E)	0.07 [−0.02, 0.16]	−0.01 [−0.11, −0.00]	0.06 [−0.04, 0.17]	0.17*** [0.08, 0.25]	−0.09 [−0.19, 0.01]	0.09 [−0.01, 0.19]	0.10* [0.00, 0.20]	−0.12* [−0.21, −0.02]	0.04 [−0.06, 0.14]
AP × E	0.11** [0.03, 0.19]	*—*	*—*	0.03 [−0.05, 0.12]	*—*	*—*	0.02 [−0.08, 0.11]	*—*	*—*
Flow	*—*	0.39*** [0.31, 0.46]	−0.28*** [−0.36, −0.19]	*—*	0.29*** [0.21, 0.37]	−0.26*** [−0.38, −0.13]	*—*	0.31*** [0.23, 0.38]	−0.21*** [−0.31, −0.10]
Age	0.03 [−0.07, 0.13]	0.09 [−0.01, 0.18]	−0.06 [−0.14, 0.03]	−0.00 [−0.09, 0.08]	0.02 [−0.08, 0.12]	−0.04 [−0.13, 0.05]	−0.00 [−0.10, 0.09]	0.05 [−0.05, 0.15]	−0.04 [−0.14, 0.06]
Relationship satisfaction	0.11* [0.01, 0.20]	−0.02 [−0.12, 0.07]	0.11* [0.02, 0.19]	0.24*** [0.14, 0.33]	0.04 [−0.06, 0.14]	0.08 [−0.03, 0.19]	0.21*** [0.10, 0.31]	0.04 [−0.06, 0.14]	0.03 [−0.08, 0.14]
Game experience	−0.01 [−0.11, 0.08]	−0.12* [−0.21, −0.02]	0.14** [0.04, 0.25]	−0.02 [−0.11, 0.06]	−0.10 [−0.20, 0.00]	0.12* [0.02, 0.22]	0.13** [0.03, 0.22]	−0.04 [−0.14, 0.05]	0.05 [−0.05, 0.15]
Win count	0.33*** [0.25, 0.40]	−0.00 [−0.07, 0.07]	−0.15*** [−0.23, −0.07]	0.26*** [0.19, 0.34]	0.08* [0.01, 0.14]	−0.12** [−0.20, −0.05]	0.17*** [0.08, 0.26]	0.08* [0.01, 0.14]	−0.16*** [−0.24, −0.07]
Simple indirect effects									
AP	*—*	*—*	*—*	*—*	0.04* [0.01, 0.07]	−0.04* [−0.07, −0.00]	*—*	0.05* [0.01. 0.08]	−0.03* [−0.06, −0.01]
E	*—*	*—*	*—*	*—*	0.05** [0.02, 0.08]	−0.04** [−0.07, −0.01]	*—*	0.03 [−0.00, 0.06]	−0.02 [−0.04, 0.00]
Conditional indirect effects									
High AP	*—*	0.05** [0.01, 0.10]	−0.04* [−0.07, −0.01]	*—*	*—*	*—*	*—*	*—*	*—*
Low AP	*—*	0.00 [−0.04, 0.04]	−0.00 [−0.03, 0.03]	*—*	*—*	*—*	*—*	*—*	*—*
*R* ^2^	0.19	0.19	0.15	0.22	0.16	0.11	0.15	0.16	0.08

*Note*: *N* = 368. Conditional indirect effects refer to the indirect effects of extraversion on LAP and HAN affect via flow experience, with autotelic personality being high (*M* + 1 *SD*) or low (*M* – 1 *SD*).

Abbreviations: HAN, high‐arousal negative; LAP, low‐arousal positive.

* *p* < 0.05;

** *p* < 0.01;

*** *p* < 0.001.

Findings were less consistent with H2 (see also Table 2). Whereas extraversion was positively associated with flow experience in mere‐presence and interactive conditions, its effect on solitary flow was not statistically significant. Nevertheless, constraining the paths across conditions to be equal did not significantly worsen the model fit (Δ*χ* ^2^[2] = 0.07, *p* = 0.071, ΔCFI = 0.002, ΔRMSEA = 0.000, ΔSRMR = 0.000). Contrary to H2, we failed to find a negative association between extraversion and solitary flow.

**FIGURE 1:**
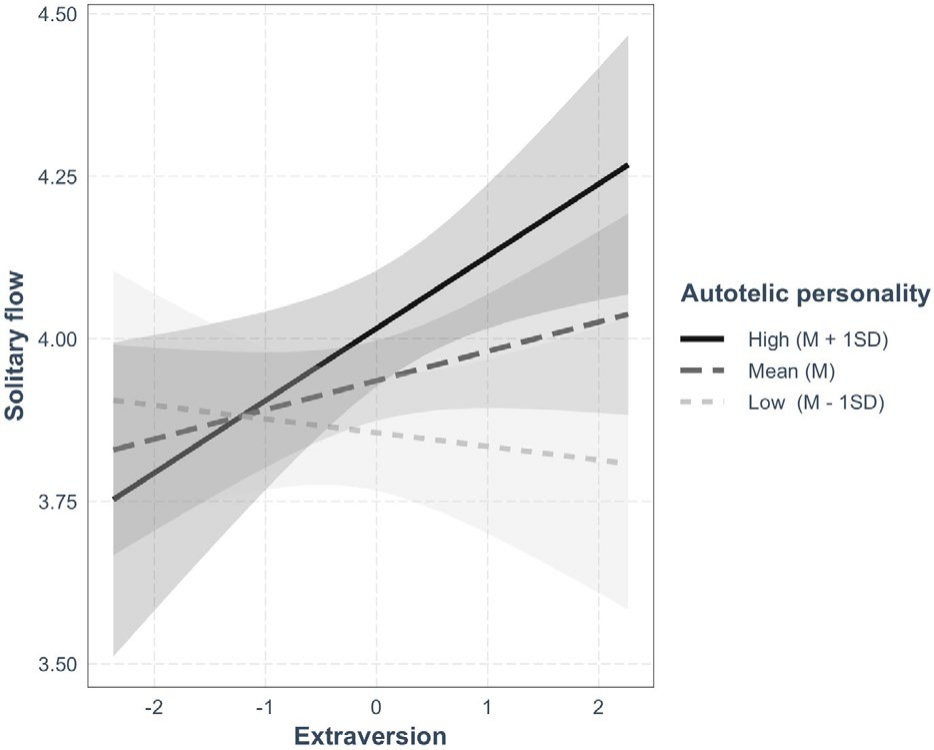
Simple slope analysis illustrating the interaction between autotelic personality (AP) and extraversion on solitary flow. Standardized betas of extraversion on solitary flow were as follows: *β* = −0.02, *p* = 0.619, 95% CI [−0.10, 0.06] for low AP; *β* = 0.05, *p* = 0.160, CI [−0.02, 0.11] for mean AP; *β* = 0.11, *p* = 0.013, CI [0.02, 0.20] for high AP.

We then examined the interaction terms between extraversion and autotelic personality (H3; see Table 2). There was a statistically significant interaction between them on flow experience in the solitary condition, but not in mere‐presence and interactive conditions. Simple slope analyses (see Figure 1) revealed that extraversion was positively associated with solitary flow when autotelic personality was high (i.e., above the mean by 1 *SD*) but not when it was low (below the mean by 1 *SD*).

### 3.2 Exploratory analyses

Finally, we explored the associations between flow experience and HAN and LAP states in three experimental conditions. Across conditions, flow experience indeed was associated with greater LAP affect and lesser HAN affect. Constraining the paths across conditions to be equal did not significantly worsen the model fit (Δ*χ* ^2^[4] = 0.06, *p* = 0.054, ΔCFI = 0.003, ΔRMSEA = 0.000, ΔSRMR = 0.001), suggesting that the relationships between flow experience and LAP affect as well as those between flow experience and HAN affect were highly similar across conditions.

Given the significant interaction between extraversion and autotelic personality on flow experience in the solitary condition and the significant relationships between flow experience and LAP and HAN affect, we conducted further analyses to estimate the conditional indirect effect of these dispositions on affect via flow experience (see also Discussion). Similar to previous analyses, the indices of conditional indirect effect were statistically significant only for the solitary condition. Whereas the indirect effects of extraversion on LAP and HAN affect (via flow experience) were significant when autotelic personality was high (above 1 *SD*), they were non‐significant when autotelic personality was low (below 1 *SD*). That is, for participants high (vs. low) in autotelic personality, greater extraversion was associated with flow experience and, in turn, greater LAP and lesser HAN affect in the solitary condition. For the mere‐presence and interactive conditions, the indirect effects of autotelic personality on LAP and HAN affect were significant, but the indirect effects of extraversion were significant on affect in the former but not the latter condition. The non‐significant indirect effect of extraversion in the interactive conditions could be due to its relatively weaker relationship with social flow, although we found no significant difference in the magnitudes of the extraversion–flow relationship across conditions.

Given that flow experience and affective states were measured simultaneously, it was necessary to evaluate the possibility of reverse causality (i.e., affective states predicting flow experience). We compared the fit of the target conditional indirect model against that of the model with LAP and HAN affect predicting flow experience (i.e., XMY vs. XYM). The latter model fit the data comparably more poorly than the target model (Δ*χ* ^2^[6] = 46.48, *p* < 0.001, ΔCFI = 0.021, ΔRMSEA = 0.015, ΔSRMR = 0.013), providing further support that greater LAP and lesser HAN affect were downstream implications of flow experiences across conditions.

## 4 DISCUSSION

We conducted an experiment investigating the impact of autotelic personality and extraversion on flow experiences when romantic partners played games alone separately (solitary), side‐by‐side with no interactions (mere‐presence), and together cooperatively (interactive). Consistent with previous findings (Baumann, 2021; Tse, Lau, et al., 2021), we found that autotelic personality predicted more intense flow experiences across conditions (H1). Contrary to our hypothesis (H2), we did not find a negative relationship between extraversion and solitary flow. Instead, extraversion appeared to have an overall positive relationship on flow experience, although such a relationship was attenuated to non‐significant in the solitary condition when autotelic personality was low (H3). Finally, although we were unable to test the deactivation hypothesis independent of affective valence (H4), flow experience across conditions was indeed associated with more LAP and less HAN states. Our findings also provided initial support to the (conditional) indirect effects of personality attributes on affective states via solitary or social flow experiences.

Our findings contribute to the identification of context‐general and context‐specific dispositions that promote solitary and social flow. Indeed, autotelic personality was associated positively with flow experience across social contexts, with very similar effect sizes, suggesting the generalizability of its facilitating effect on both solitary and social flow. We acknowledge the conceptual similarity between autotelic personality and flow experience, especially as the former is often defined as the facilitator of flow experience in the literature (e.g., Nakamura & Csikszentmihalyi, 2002). That said, our findings suggest that (a) they are more likely to be two separate yet positively associated factors rather than one psychological construct, based on the model comparison between two latent models; (b) flow experience is subject to the influence of situational factors, such as the nature of the task, beyond autotelic personality, given the small effect sizes of their relationships (see Table 2); and (c) flow experience (vs. autotelic personality) is correlated more strongly with LAP and HAN affect measured in the corresponding situation, according to the zero‐order correlation coefficients (see Table S1). Taken together, the findings support the discriminant validity of autotelic personality and flow experience, and the former appears to be a context‐general, yet not dominating, facilitating factor of flow experience across social contexts.

The findings on extraversion are less clear‐cut. On one hand, studies that measure global flow experience suggest an overall positive effect of extraversion on promoting flow experience (see Peifer et al., 2022 for a review). On the other hand, when social and solitary flow are differentiated and measured separately, extraverts appear to enjoy more intense and frequent flow experiences in social activities, whereas introverts perceive solitary activities to be more flow‐conducive than interactive activities (Liu & Csikszentmihalyi, 2021). Our findings revealed that extraverts appear to find the solitary condition no less flow‐conducive than introverts. Additionally, the overall positive extraversion–flow experience relationship was attenuated in the solitary condition, suggesting some degree of context specificity of such a relationship.

Besides sociability and assertiveness, other facets commonly under extraversion include energy/activity level (Soto & John, 2017), excitement‐seeking, and positive emotionality/enthusiasm/liveliness (Costa & McCrae, 1992; John & Srivastava, 1999; Lee & Ashton, 2008). These facets appear to overlap with flow experience characterized by greater enjoyment and motivation in an activity (Csikszentmihalyi, 1990), enabling extraverts to enjoy the experimental task even if it lacks the social component in which they typically thrive. That said, simple slope analyses revealed that this positive association between extraversion and solitary flow was only significant when autotelic personality was high; extraversion was neither a promoting nor inhibiting factor of solitary flow when autotelic personality was low. We posit that low autotelic personality reflects a weaker ability to transform and enjoy activities that are perceived as boring, tedious, or trivial (Baumann, 2021; Tse, Nakamura, et al., 2021). As such, this negates the tendency of extraverts getting absorbed and excited in activities and, hence, results in the observed interactive effect on solitary flow. Given that we only used a flow‐conducive game in this experiment, future studies can systematically vary the challenge level of the task to examine its impact on the extraversion‐solitary flow relationship.

Studying solitary flow generates insights into the essence of positive solitude (see also Ost Mor et al., 2021). We found that both solitary and social flow experiences were associated with greater LAP and lesser HAN affect. Although we did not originally intend to include HAN affect in our investigation, the negative flow–HAN affect relationship is consistent with the literature, as studies have suggested how flow experience helps mitigate anxiety and worries in highly stressful environments, such as during uncertain waiting periods and the COVID‐19 pandemic (Rankin et al., 2019; Sweeny et al., 2020).

Additionally, the highly similar effect sizes across conditions further suggest that when activity type is experimentally controlled, the affective experiences of flow across social contexts are likely to yield little difference. In this experiment, we employed a challenging (yet achievable) board game as the experimental task, as opposed to previous solitary studies with tasks that were relatively passive, such as refraining from doing any activities, reading leisurely, or thinking about something alone (e.g., Nguyen et al., 2018). We speculate that whether solitude has a unique downregulation effect on arousal above and beyond social interactions may depend on the nature of the solitary activity. Playing a challenging game is likely to maintain a certain level of physical or affective arousal, regardless of the social contexts. This may account for our inability to find stronger LAP and HAN associations with solitary flow than with social flow. That said, we acknowledge that our findings were unable to directly address the downregulation hypothesis, especially when the data did not cover the full spectrum of the affective circumplex. Nevertheless, the conditional indirect effects suggest that a combination of high extraversion and high autotelic personality may contribute to positive solitary experiences, with a person's ability to get absorbed and engaged in the solitary activity being one of the potential underlying mechanisms. Acknowledging the multiple positive solitude profiles across individuals (Ost Mor et al., 2021; Yu et al., 2022), future studies can explore how different dispositions may be associated with solitary experiences through different psychological mechanisms.

### 4.1 Strengths, limitations, and future directions

The experimental design of this study enabled us to empirically evaluate the differential relationships of personality and flow experience in solitary and social conditions with high experimental control. The inclusion of the mere‐presence condition as an active control also helped identify whether the relationships differed due to the absence of social interactions or the physical absence of others in the same space, which have been two popular yet commonly conflated definitions of solitude in the literature (Campbell & Ross, 2022). In this study, we found that the indirect effects of extraversion on LAP and HAN affect were significant in the mere‐presence condition but not in the interactive condition, suggesting the former is unlikely a conceptual “middle‐ground” between complete solitude and social interaction. This interesting finding warrants future investigation to evaluate the uniqueness of such a social context beyond a simple solitude–social interaction dichotomy.

However, this study also had its limitations. First, our study only compared the solitary condition with those that involved participants' romantic partner. Many productive and leisure activities, such as teamwork and team sports, frequently involve more than one social target who often does not have a strong social tie with the person. Especially in the mere‐presence condition, the recent literature suggests potential conceptual distinctions between private solitude (that includes being physically alone but also sharing a private space with people of “your own kind”; Weinstein et al., 2022, p. 1668) and public solitude with the presence of acquaintances or strangers. Future research can investigate not only the experiential differences in interactions with different social partners (e.g., Merz & Huxhold, 2010) but also the differences when these social partners are merely in the same space.

Second, although using the same experimental task (Perfection™) across social contexts eliminate potential confounds and enable considerably fairer comparisons, people often have different activity profiles in solitude or in social interactions (e.g., Hipson et al., 2021). Instructing participants to play the Perfection™ game (without alternatives) and putting them in different experimental conditions in random order (i.e., social context not self‐selected) may have undermined their sense of autonomy, which is an important contributing factor of positive solitary experience (Nguyen et al., 2018; Tse et al., 2022). Future studies can test whether allowing participants to engage in a flow‐conducive activity of their choice may further enhance their solitary experience.

Despite these limitations and our inability to test the original Hypothesis 4 fully due to experimenters' errors, our findings illustrate that personality traits, specifically autotelic personality and extraversion, contribute to a person's solitary and social flow, with those who are simultaneously high in both dispositions appear to enjoy solitary flow the most. While people may spend their time alone on various activities, getting absorbed and engaged in a solitary activity can be one viable way for some individuals to enjoy solitude.

## AUTHOR CONTRIBUTIONS

Dwight C. K. Tse: Conceptualization, data curation, formal analysis, investigation, methodology, project administration, supervision, writing‐ original draft, writing‐ review & editing. Ayodele Joseph: Conceptualization, investigation, methodology, writing‐ original draft, writing‐ review & editing. Kate Sweeny: Conceptualization, data curation, methodology, resources, project administration, writing‐ original draft, writing ‐ review & editing.

## CONFLICT OF INTEREST STATEMENT

The authors have no conflicts of interest to declare.

## ETHICS STATEMENT

This study was reviewed for ethics approval by the Internal Review Board at the University of California, Riverside.

## Supporting information


Table S1.

## Data Availability

All study data and data analysis codes are available at https://osf.io/pqx3z/ (also including the Stage 1 Registered Report) and in the special issue's Open Science Framework (OSF) repository.

## Endnote

^1^
Although we stated our inclusion criteria explicitly in the recruitment materials, four participants reported their relationship length to be shorter than 6 months. Unsure whether they had mistaken the unit as years or not, we retained these records in our data. Sensitivity analyses revealed no differences in our statistical conclusions with or without these cases.
