# Supplementary material for: Alone but flowing: The effects of autotelic personality and extraversion on solitary flow
Source: J Pers. 2024 May 10;93(1):67–80. doi: 10.1111/jopy.12938 (PMC11705519; doi:10.1111/jopy.12938)
Supplement: Supplementary file 1 — Table S1. [file JOPY-93-67-s001.docx]

**Supplemental Table S1**

*Means, Standard Deviations, and Correlations with Bootstrapped 95% Confidence Intervals*


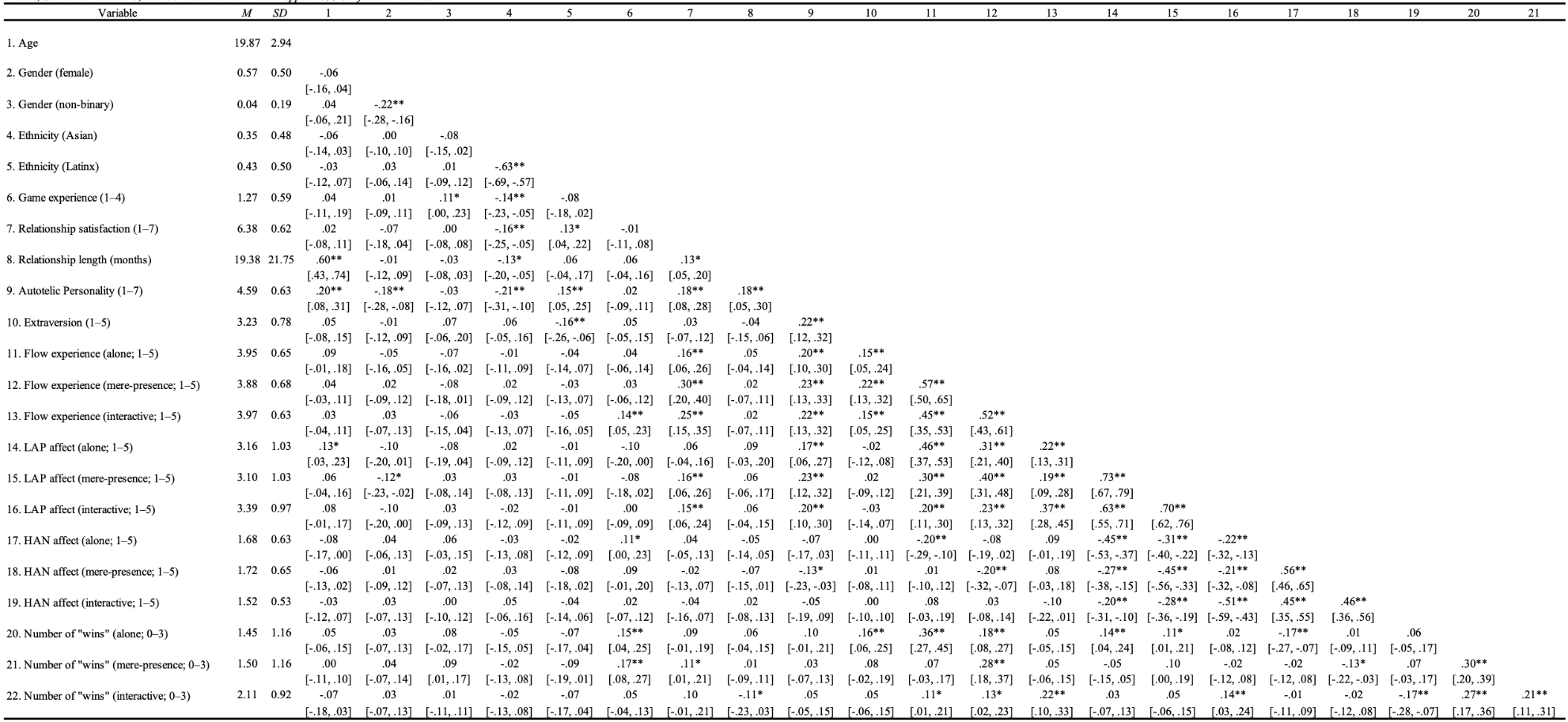


*Notes*. * *p* < .05. ** *p* < .01.
